# A malaria knowledge, attitudes and practice survey in a rural community in Guinea

**DOI:** 10.1186/s12936-022-04357-6

**Published:** 2022-11-14

**Authors:** Nirmal Ravi, Erin Holsted, Barclay Kadiebwe, Abigail Salthouse, Amer Sattar

**Affiliations:** eHealth Africa, 4-6 Independence Rd, Kano, Nigeria

**Keywords:** Malaria, Republic of Guinea, Africa, Knowledge, attitudes, and practice (KAP) survey, Water, Hygiene, and sanitation practices, Diarrhoea, Childhood, Healthcare access

## Abstract

**Background:**

Malaria is the top public health problem in the Republic of Guinea, with more than 4 million cases and 10,000 deaths in 2021 among a population of approximately 13 million. It is also the second highest cause of death there. The purpose of this quantitative survey in a rural area of Guinea was to understand knowledge, attitudes, and practices (KAP) about malaria and to assess water and sanitation practices among community members.

**Methods:**

In 2016, the authors conducted a cross-sectional household survey in Timbi-Touni, Guinea using community workers. The survey included respondent demographic characteristics, malaria knowledge, child health, water and sanitation, and health services access. Malaria knowledge and sleeping under bed nets were the primary outcome variables and multiple logistic regression was used to determine odds ratios.

**Results:**

Majority of the respondents were women (89.41%) and had never been to school (71.18%). Slightly more than half the children were reported to have ever had malaria and 45% reported to have ever had diarrhoea. There was no statistically significant association between gender or level of education and malaria knowledge. Eighty six percent of respondents had received a free bed net during national campaigns and 61% slept under a bed net the night before the survey. Knowing mosquitoes to be the cause of malaria and receiving free bed net were significantly associated with sleeping under a bed net. There was no statistically significant association between drinking water source and malaria or diarrhoea.

**Conclusions:**

Both malaria and diarrhoea were considered to be serious illnesses for adults and children by nearly all respondents. Receiving free bed nets and having correct knowledge about malaria were the greatest predictors of sleeping under a bed net. Insights from this detailed KAP survey—such as focusing on radio to transmit malaria prevention information and reinforcing free malaria treatments—can guide policy makers and practitioners who design and implement malaria control and prevention measures in Guinea.

## Background

Malaria is a life-threatening disease that poses a health threat to nearly half of the global population. The World Health Organization (WHO) Africa Region continues to endure the largest burden of this illness. In 2018, 93% of malaria cases and 94% of malaria deaths occurred in sub-Saharan Africa [1]. About 24 million children were estimated to be infected in 2018 in sub-Saharan Africa.

The Republic of Guinea is situated in the Gulf of Guinea in West Africa bordered by Sierra Leone, Liberia, Senegal, Guinea-Bissau, Mali, and Ivory Coast. Guinea’s population of approximately 13 million inhabitants is considered to be at risk of this vector borne illness that has year-round transmission, with the peak transmission occurring during the July through October rainy season [2]. According to the National Strategic Plan of Guinea, malaria remains the number one public health problem accounting for a third of all patient visits [2]. The routine surveillance system in Guinea reported 992,146 cases of malaria and 867 deaths in 2016 [2]. Guinea is one of the countries with the highest percentage of severe anaemia among children aged under 5 years who were positive for malaria, and it is the second highest leading cause of death for all age groups [3] A recent national household survey of children aged 6 months to 9 years showed malaria prevalence between 44 and 61% with rural areas showing high prevalence [4].

National Malaria Control Programmes and donor agencies spent nearly $2.7B worldwide in 2018 for malaria control and eradication [1]. In 2018, Guinea received $12 M from The Global Fund and $15 M from USAID [1]. Guinea had two mass campaigns for distribution of long-lasting insecticidal nets (LLIN) in 2013 and 2016 [2]. LLINs are also given to pregnant women during prenatal care visits and 59% of the population had access to insecticide-treated nets in 2016 [1]^)^. Guinea adopted a new National Strategic Plan in 2018 to reduce malaria morbidity and mortality by 75% of 2016 level and achieve pre-elimination by 2022 [2].

Despite such intensified efforts by donors and the National Malaria Control Programme, the annual number of malaria cases continued to increase [1]. National level data is extremely important for monitoring progress towards malaria elimination and informing public policy. However, local surveys can inform intervention design by providing the perspective of those facing the disease. Research of this kind is sparse in Guinea. However, previous studies in similar contexts have documented relationships between knowledge about malaria and several factors [5–7]. Low socioeconomic status and lack of education have been consistently associated with an overall lack of knowledge about malaria in the literature [5].

According to UNICEF, one-third of Guineans drink unsafe water which is the root cause of many health conditions and a large contributor to child mortality [8]. A 2020 study sought out to investigate if improved drinking water and sanitation conditions were associated with a decreased risk of malaria infection among children under the age of five in sub-Saharan Africa. Unprotected drinking water and lack of use of sanitation facilities were associated with increased malaria risks [9].

The purpose of this quantitative survey in a rural area of Guinea was to understand knowledge, attitudes and practices (KAP) about malaria and to assess water and sanitation practices among community members. The information gathered can further inform and guide Guinea’s malaria control strategies towards the goal of pre-elimination by 2030.

## Methods

### The KAP survey

**Fig. 1:**
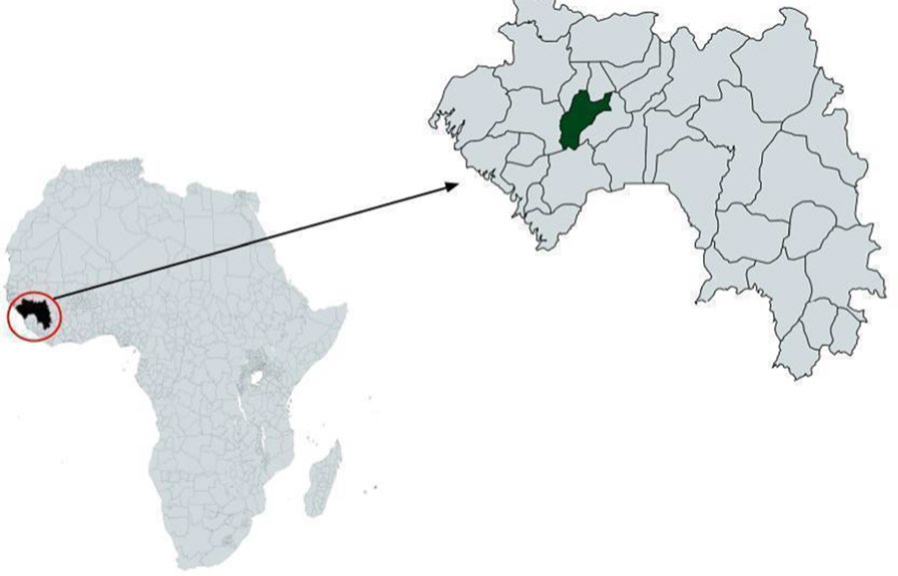
Guinea and the study site of Pita prefecture

A cross sectional household survey was conducted in 2016 in the sub-prefecture of Timbi-Touni (population 20,719), Pita Prefecture in the Republic of Guinea (See Fig. 1). Timbi-Touni was chosen for the survey as part of a larger community health improvement program implemented by eHealth Africa.A sample size of 300 households was calculated based on the population and estimated number of households, and assuming a 20% non-response rate. The interviews were done by community workers and those responsible for the local government health posts. The interviewers attended a two-day training course in order to understand the questionnaires, master the collection tool, understand the informed consent script, understand the importance of ethics and confidentiality, and know the method of randomization.

They spoke fluent French, as well as the local language Pular in order to communicate well with the participants. Community workers used mobile phones to record interview responses using an ODK (Open Data Kit) tool implemented by eHealth Africa.

The questionnaire was administered to adult men and women who were present in the household when the interviewer visited. Investigators asked if the house visited had at least one child under the age of five. If the household did not have any child under five, the investigator thanked the individual and went to the next house. In each village within each district, the same number of households were visited. For villages with a health facility, household No. 1 was chosen as the one closest to the health facility; for villages without a health facility, proximity to the office of the village chief was considered. Then, the following households were identified by a zigzag movement. There was no marked variation in households within the survey area due to its rural nature.

### Selection of variables

The questionnaire was structured to take approximately 50 min and was composed of 49 multiple-choice questions. The interview was structured into six distinct sections: (1) respondent demographic characteristics, (2) child health, (3) child health promotion related to malaria knowledge, (4) water and sanitation, (5) health services access, (6) interviewee opinions concerning communication of malaria information and access to prevention methods.

### Data analysis

Data analysis was done using SAS Version 9.4 and Stata 15. Descriptive statistics were used to visualize the data using graphs created using Microsoft Excel. The Chi-square test was used to determine differences between groups except to test association with level of education in which case Fisher’s exact test was used due to sparse data. Multiple logistic regression was used to determine adjusted odds ratios.

### Ethical consideration

The survey was only conducted after informed verbal consent of each participant was obtained. The questionnaire was anonymous and confidential. All responses were collected and stored electronically using password protected software. Ethics approval was obtained from the Guinea National Ethics Committee for Health Research (96/CNERS/16).

## Results

### Study population characteristics

**Fig. 2:**
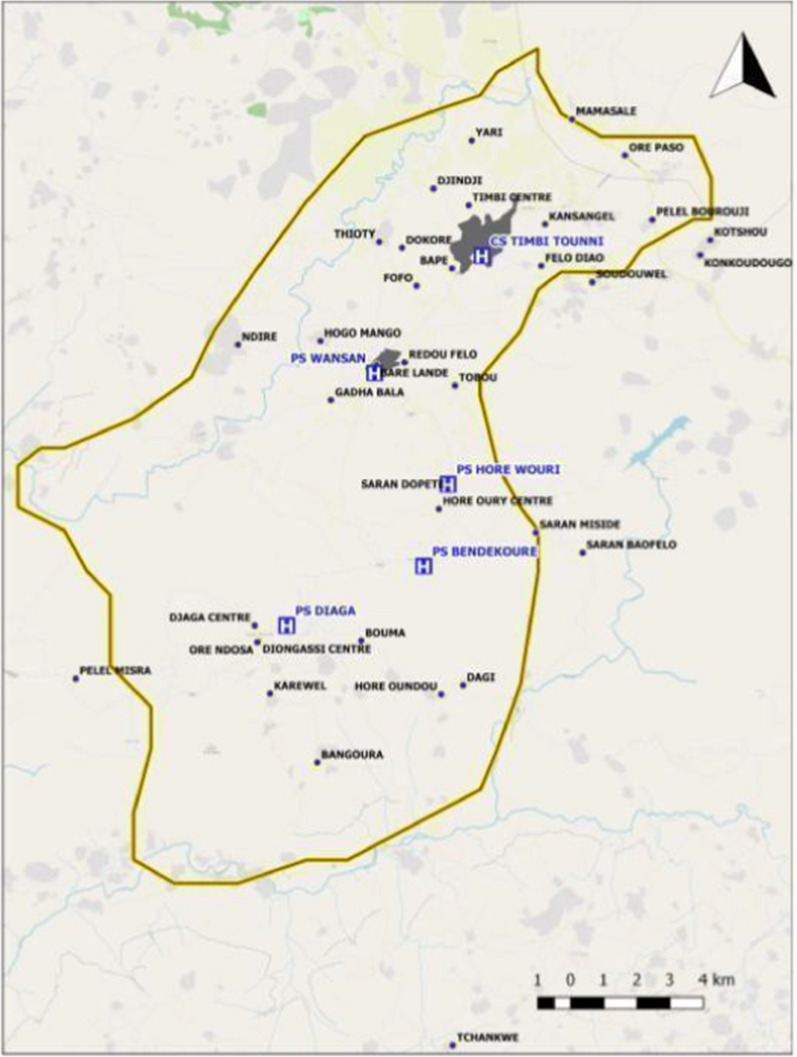
Timbi-Touni with surveyed villages

A total of 406 respondents/completed questionnaires from the interviewed households were used in the analysis of the survey data. Eight households did not consent to the survey and the questionnaires were not completed. A map of the health post locations that were surveyed is shown in Fig. 2.

The majority of respondents were female (89.41%) and had never been to school (71.18%), followed by less than 6 years of education (22.17%), more than 10 years (5.42%), and more than 15 years (1.23%) of education (Table 1). The average number of members in the household was 5.09 ± 2.56. The average number of children under five in a household was 1.21 ± 0.43.

**Table 1:** Socio-demographic characteristics of survey participants

Characteristics	n	%
Gender		
Female	363	89.41
Male	43	10.59
Level of education		
None	289	71.18
Less than 6 years	90	22.17
More than 10 years	22	5.42
More than 15 years	5	1.23

The total number of children under the age of 5 included in this analysis is 493. The average age of the children was 28.83 ± 17.36 months and 51.93% were female. Fifty four percent of the children in the survey were reported to have been born in a hospital.

### Child health

**Fig. 3:**
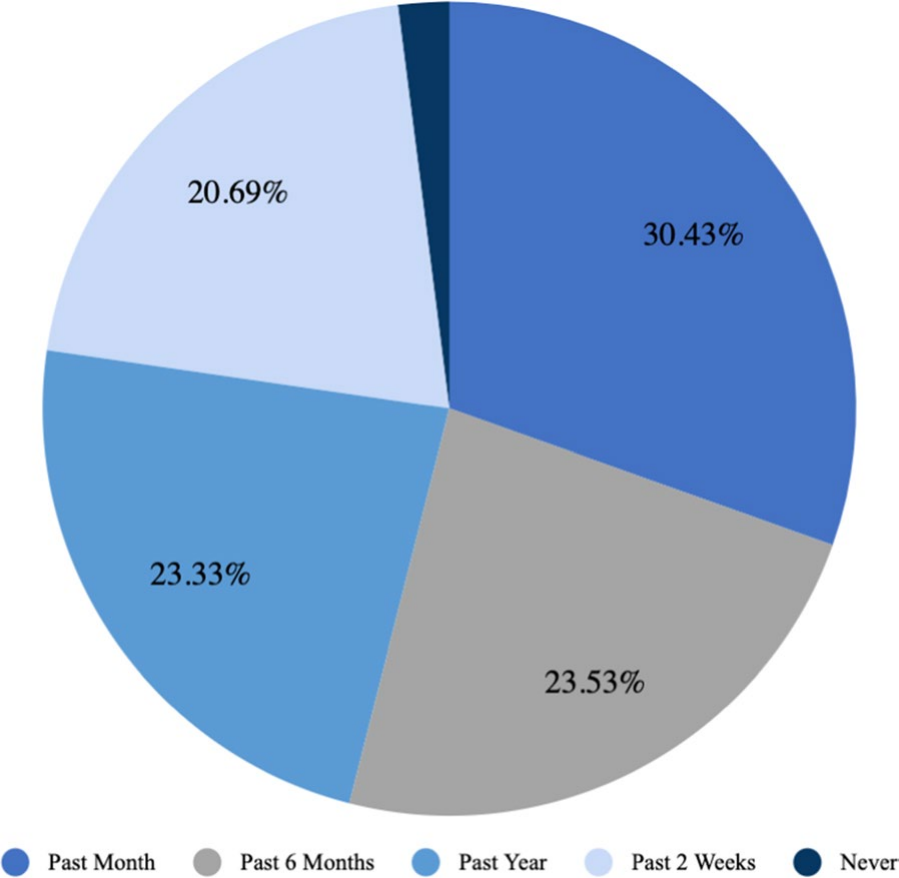
Last time respondents went to a health post

**Fig. 4:**
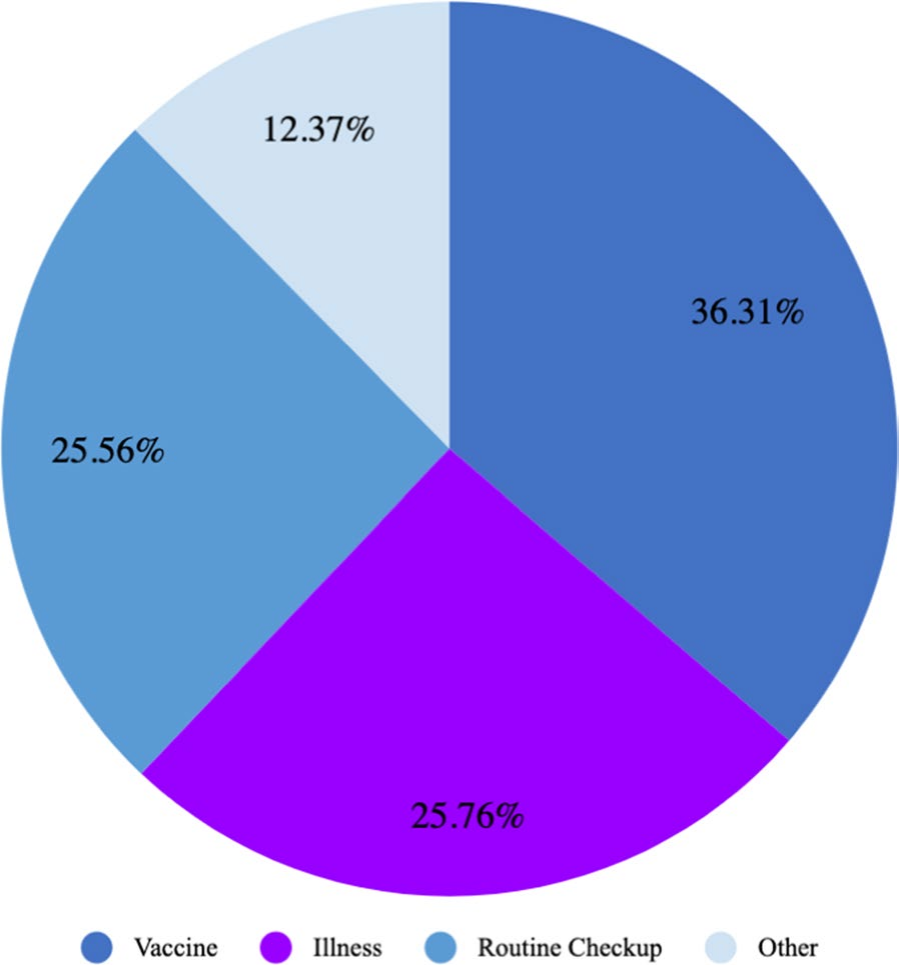
Reasons for a child’s clinic visit

The respondents, who were caregivers for the children in this survey, were asked if their children had ever had malaria or diarrhoea. It was reported that 59.84% of children had ever had malaria and 45.44% of children had ever had diarrhoea. Of the children who had ever had malaria, their caregivers reported that 52.54% of them slept under a bed net the previous night. Additionally, the respondents were asked to report the last time that the child was taken to a health post and the reason for that clinical visit. A plurality of children were taken to a health facility in the past month (30.43%), followed by in the past six months (23.53%), past year (23.33%), past two weeks (20.69), and never (2.02%) as shown in Fig. 3. The primary reason for the clinical visit was to get a vaccine (36.31%). Other reasons were as follows: because the child was very ill (25.76%), for a routine checkup (25.56%), and other reasons (12.37%) (Fig. 4).

### Knowledge of malaria transmission and practices

**Fig. 5:**
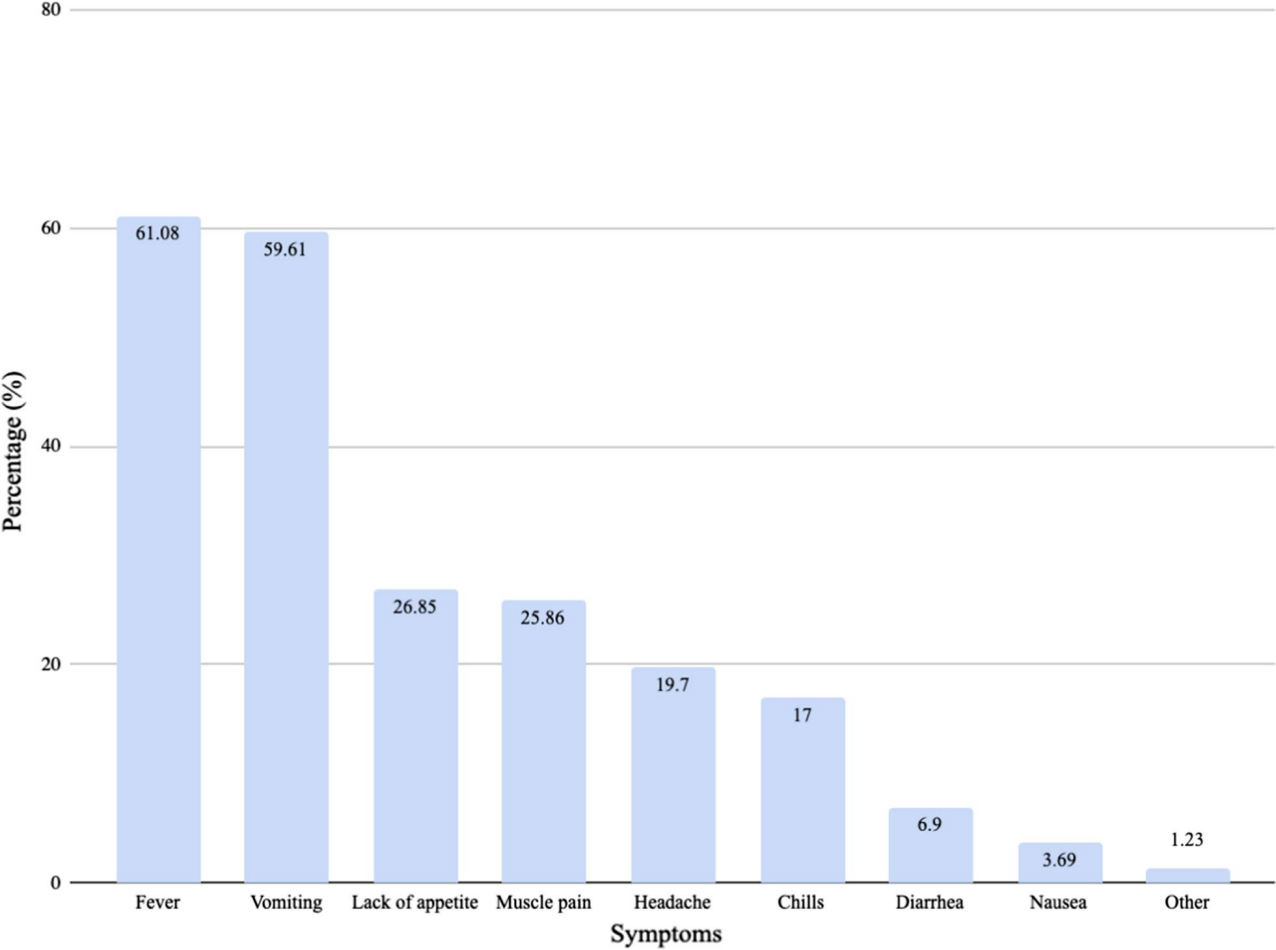
Malaria symptoms identified by respondents

**Fig. 6:**
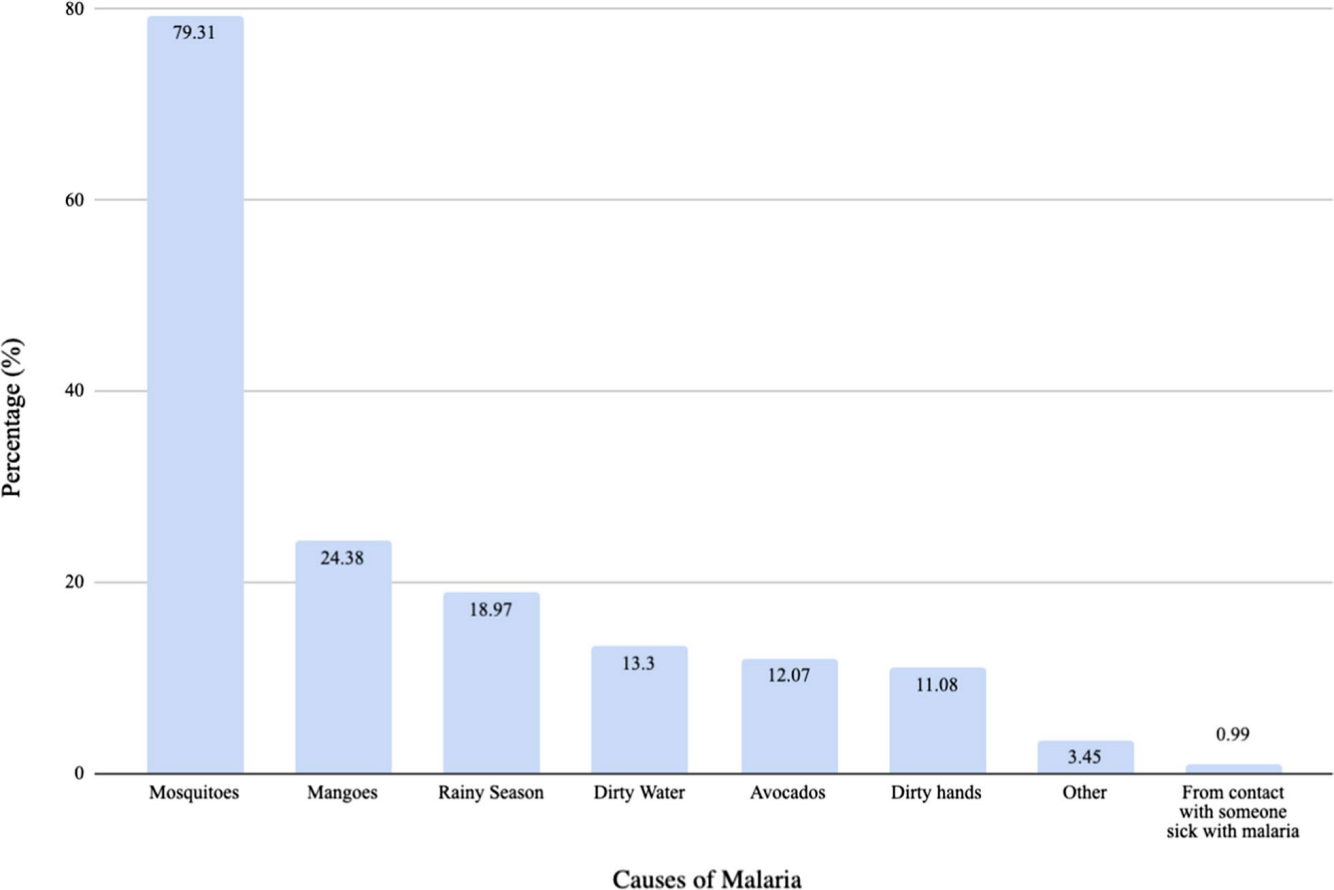
Causes of malaria identified by respondents

Participants were asked to provide the common signs that an infant or child has malaria, and they were also asked to report in which way malaria was transmitted. These results are shown in Figs. 5 and 6, respectively. The correct answer to the question “What are the causes of malaria?” served as the indicator for malaria knowledge. The majority of participants mentioned that malaria was transmitted by mosquitoes (79.31%). However, only 39.66% correctly stated that mosquitoes were the only cause of malaria. There was no difference in malaria knowledge between male and female respondents (p = 0.10). Additionally, there was also no difference in malaria knowledge amongst individuals who had completed various levels of education when using the Fisher’s exact test (p = 0.44). There was borderline statistical significance between malaria knowledge and having received malaria prevention information (p = 0.06).

With regards to malaria prevention practices, 85.71% of participants had received a bed net for free during a national campaign and 60.69% responded that they slept under a bed net the previous night.

Seventy six percent of those with correct malaria knowledge slept under a bed net the previous night compared to 50.61% of those who did not have correct malaria knowledge (p < 0.001). Similarly, 70% of those who received a free bed net slept under a bed net the previous night compared to 5% of those who did not receive a free bed net (p < 0.001). Sixty five percent of those who received malaria prevention information slept under a bed net the previous night compared to 43% who did not receive malaria prevention information (p = 0.004). There was no statistically significant association between level of education and sleeping under a bed net the previous night when tested using the Fisher’s exact test (p = 0.49).

Multiple logistic regression showed that the odds of sleeping under a bed net for people with correct malaria knowledge was 3.64 times the odds of those without correct malaria knowledge. Odds of sleeping under a bed net for those who received free bed net was 46.89 times the odds of those who did not receive free bed net (Table 2). The association between receiving malaria prevention information and sleeping under a bed net was not statistically significant after adjusting for confounders (Table 2).

**Table 2:** Adjusted odds ratios of sleeping under bed net

	Adjusted odds ratio (95% CI)	p-Value
Malaria knowledge		
Incorrect	1.0	
Correct	3.64 (2.15–6.16)	0.00
Malaria prevention information		
Not received	1	
Do not remember	1.30 (0.49–3.43)	0.59
Received	1.96 (0.97–3.94)	0.06
Free bed net		
Not received	1	
Received	46.89 (13.93–157.79)	0.00

### Water, sanitation, and hygiene practices

**Fig. 7:**
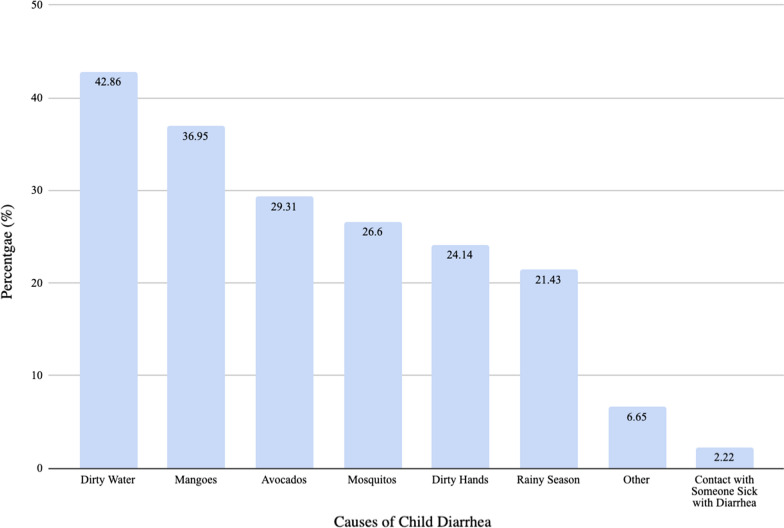
Causes of childhood diarrhoea identified by respondents

**Fig. 8:**
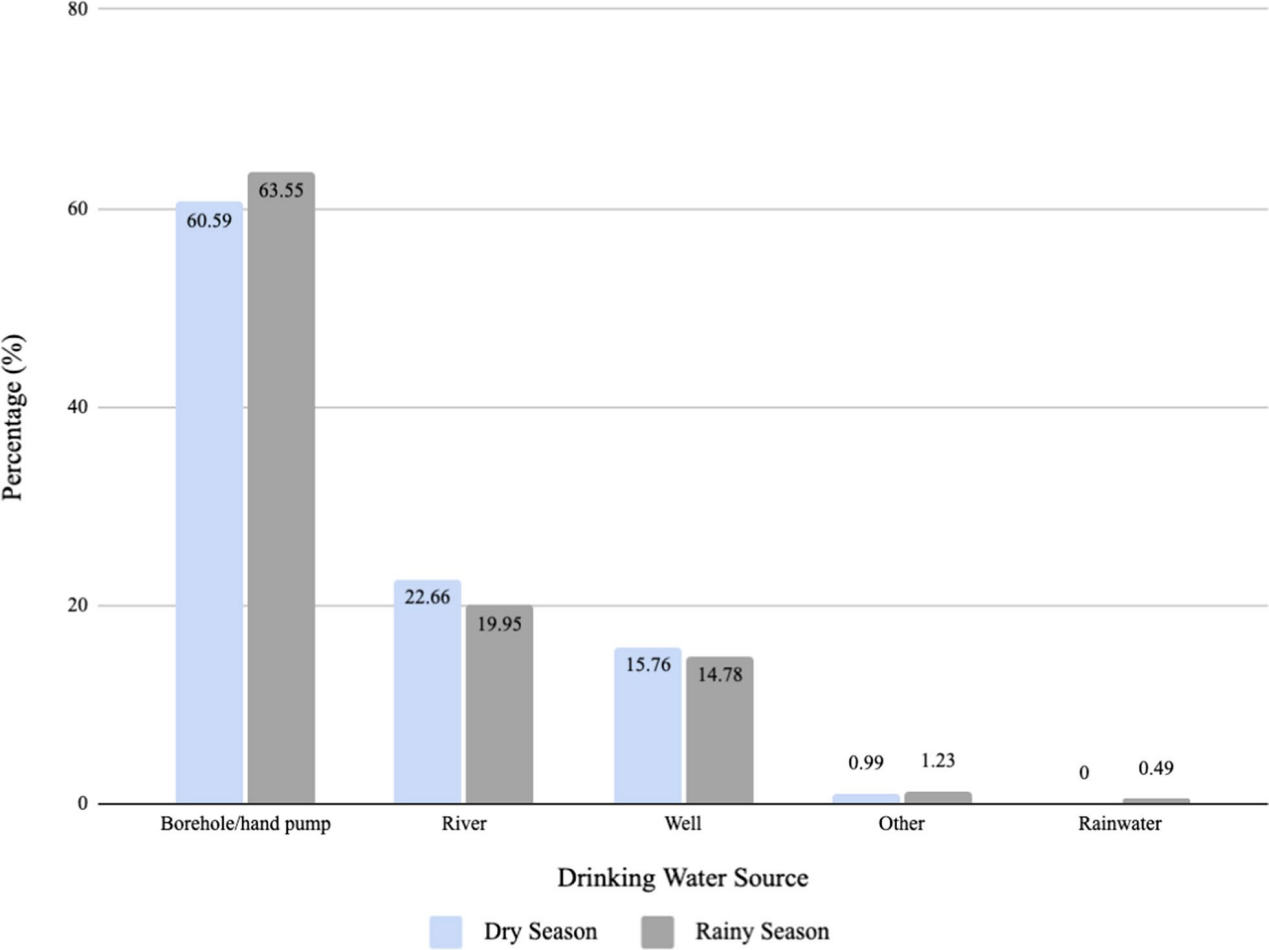
Respondents’ water source

In order to collect information on people’s knowledge of childhood diarrhoea, participants were asked to provide all the reasons they believed to be causes of diarrhoea. A plurality of respondents attributed childhood diarrhoea to dirty water (42.86%). Other reasons reported are shown in Fig. 7. The majority of respondents indicated that their source of drinking water in the rainy season and the dry season was a borehole/hand pump, 63.55% and 60.59% respectively (Fig. 8). Only 3.94% of individuals reported that they boiled water before consuming it. There was no statistically significant association between the source of drinking water during the rainy season (p = 0.49) or dry season (p = 0.19) and malaria infection in children. Similarly, there was no statistically significant association between the source of drinking water during rainy season (p = 0.49) or dry season (p = 0.84) and diarrhoea in children.

The participants were also asked a series of questions regarding hand washing practices. On average, the respondents washed their hands 4.32 ± 4.40 times per day, mostly after using the toilet (43.35%), before eating (33.50%), and before praying (9.85%). The respondents were asked if they had soap in their household, and if so, they were asked to show the interviewer the soap. Ninety seven percent of the respondents said that they have soap in their household, however, only 74.94% of such individuals were able to produce the soap during the interview.

For the children who ever had malaria, their caretakers washed their hands with soap on average 4 times per day. For the children who never had malaria, their caretakers washed their hands with soap on average 5 times per day. This difference in handwashing frequency was statistically significant (p = 0.0008). Similarly caretakers whose children never had diarrhoea washed their hands more frequently compared to those whose children ever had diarrhoea (p = 0.046).

### Health services access

**Fig. 9:**
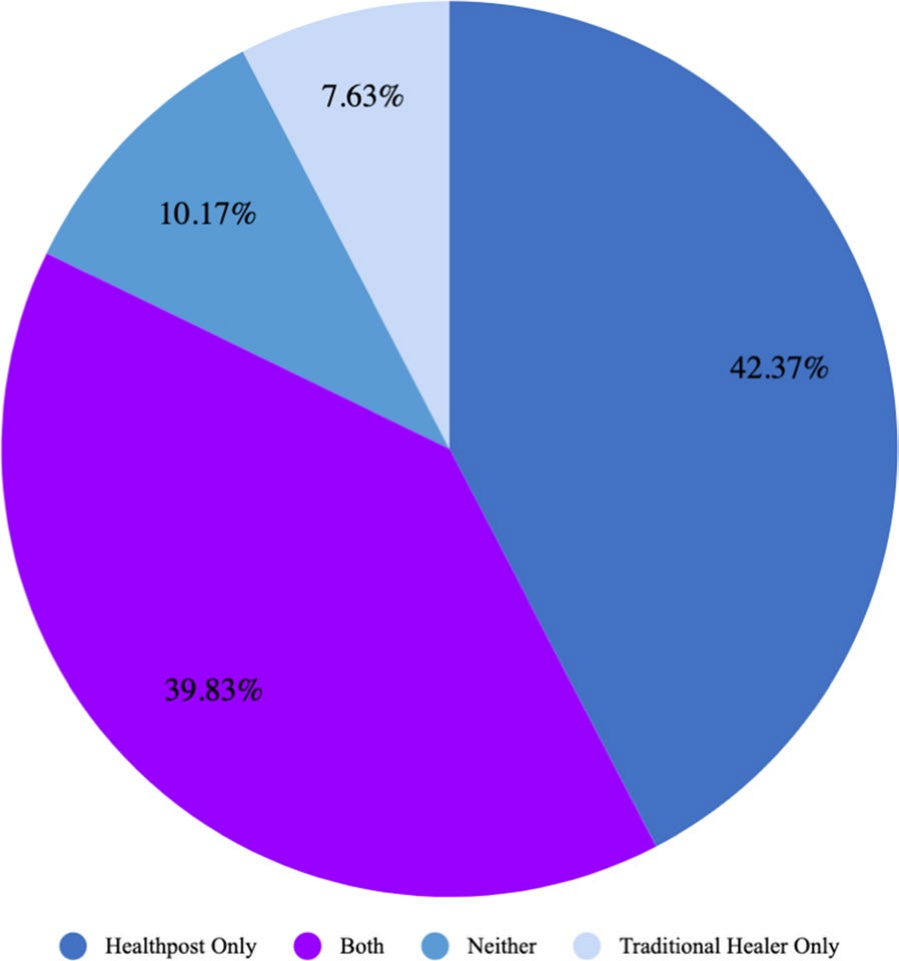
Type of health care utilized by respondents

Interviewees were asked if they had any illness or injury in the past year that kept them from doing their usual work and other activities for at least one full day. If they answered yes to this question, they were asked a series of questions related to health-seeking behaviour. Twenty nine percent of respondents answered “yes” to having an illness or injury in the last year that prevented them from doing their usual activities. Of these individuals, 7.63% sought care from a traditional healer only, 42.37% sought care from the health post, and 39.83% accessed both forms of healthcare providers (Fig. 9). Ninety-one percent of those who accessed a traditional healer bought herbal medicines. The majority of individuals bought medicines from their health post (84.62%), compared to buying them at a pharmacy. Almost all respondents (94.95%) indicated that they were satisfied with the care they received at the health post or hospital they visited.

**Fig. 10:**
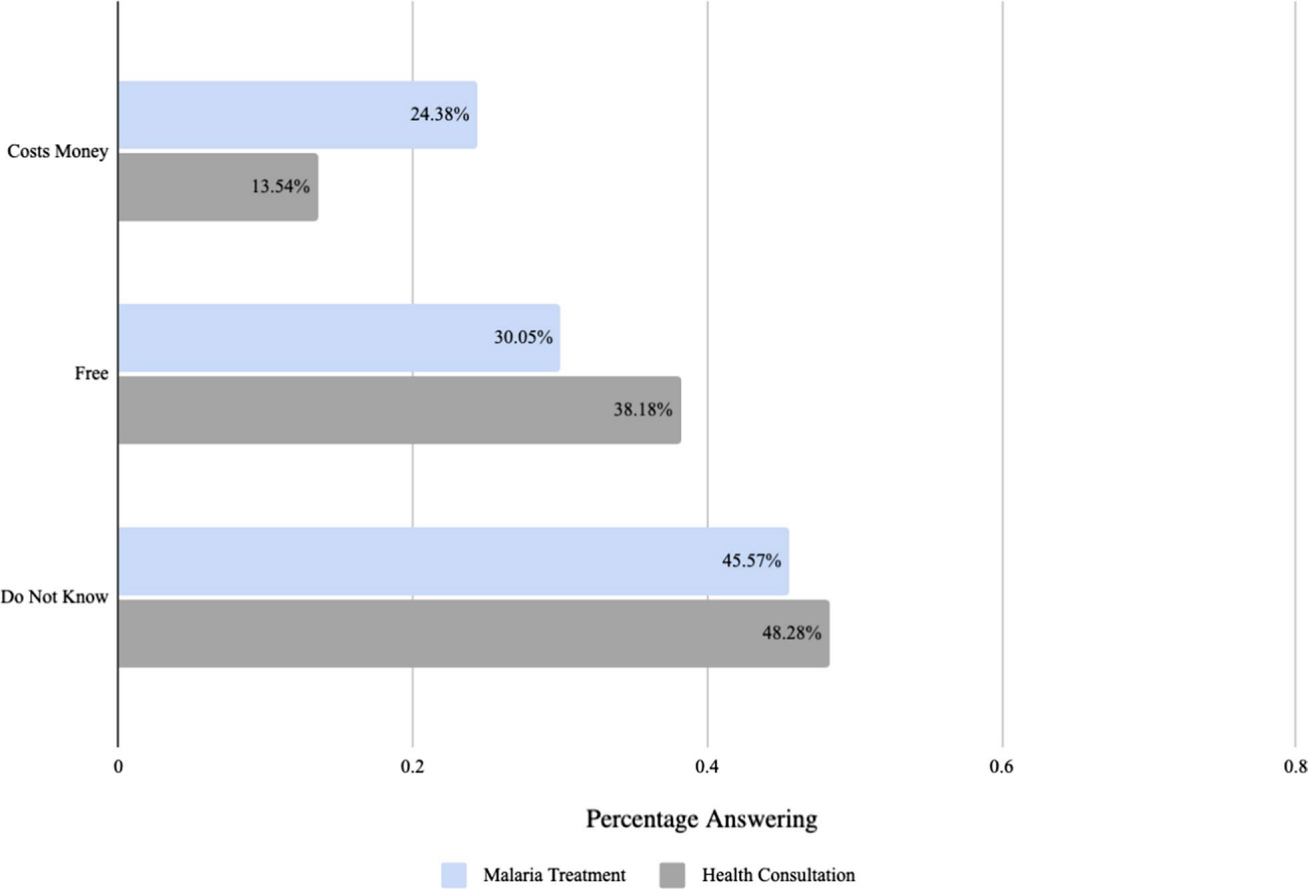
Cost of malaria treatment and health consultation

All interviewees were asked about the cost of malaria treatment at their local health post. 30.05% said that it was free, 45.57% did not know the cost, and those who indicated it costed some amount of money reported an average of 76,786 Guinean Francs (GNF) (USD 8.45). Additionally, when asked to report the cost of a consultation with a clinician at a health post, 38.18% said that it was free, 48.28% did not know the cost, and those who indicated it cost some amount of money, reported an average of 14,727 GNF (USD 1.62). These results are shown in Fig. 10.

**Fig. 11:**
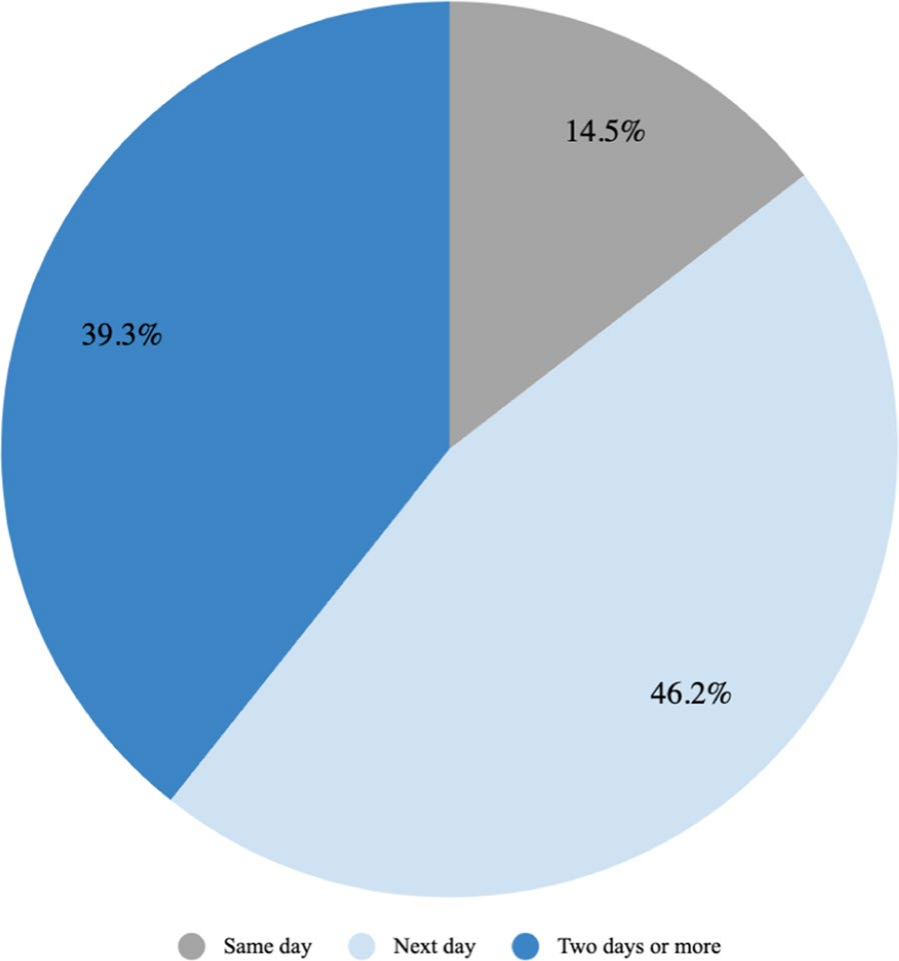
Time taken to seek treatment

A large number of respondents, 91.63%, stated that there has been a time when they or someone else in their household was very ill and thought they should go to a health post but decided not to go for medical care. Over half the participants indicated that it takes at least 30 min to travel to a health facility, and 45.92% reported that it takes longer than 1 h. With regards to those individuals who indicated that they had an illness or injury in the last year that inhibited them from doing usual activities, 39.32% reported that it took two days or more before they sought treatment or medicines after noticing the illness, followed by 46.15% who sought treatment the next day, and 14.53% the same day (Fig. 11). This serves as an indicator for delay in seeking care.

### Disease perception

A series of questions were asked to gain understanding of health-related opinions held by survey respondents.

### Illness

The vast majority of the respondents thought that both malaria and diarrhoea were serious illnesses for adults and children. With regards to malaria, 98.28% of respondents thought it to be a serious illness in adults and 99.01% thought so for children. For diarrhoea, 97.54% of respondents thought it to be a serious illness in adults, and 98.77% held this opinion in relation to children.

### Allopathic medicine

As previously mentioned, many of the respondents in this survey sought the care of a traditional healer and bought herbal medicines. However, when they had malaria, 44.09% of individuals said they preferred pills as treatment, and 44.34% preferred an injection. For those that preferred an injection, it was for the following reasons: they are stronger (60.1%), they are faster (37.16%), and other reasons (2.75%).

### Health information

**Fig. 12:**
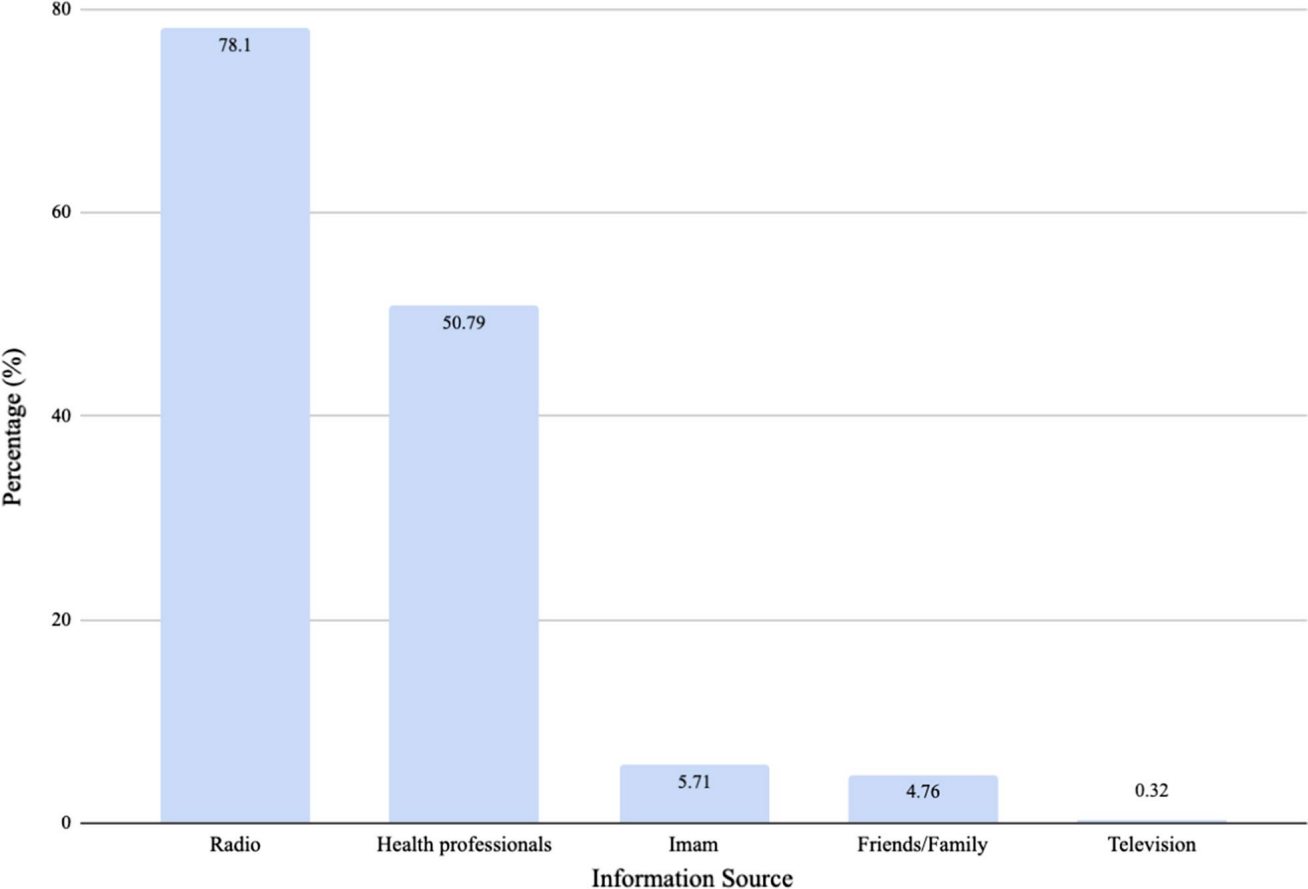
Trusted sources of health information

Participants were asked to state their preferred and most trusted source of health information. Seventy-eight percent of individuals trusted radio for their health information. Other sources of information are shown in Fig. 12.

## Discussion

KAP surveys are important for gaining insight into the misconceptions or misunderstandings that may serve as obstacles to prevention and treatment interventions that public health programs aim to implement [10]. It has been reported that childhood malaria prevalence is influenced by a complex interplay of socioeconomic, cultural, educational, geographical and environmental factors [11–13]. Because malaria is endemic to Guinea and all inhabitants are at risk of infection, it is imperative that local communities are assessed to identify gaps in knowledge so that resources and programmes are allocated effectively. There was an overall paucity of scientific literature on malaria from the Republic of Guinea, especially as it relates to water, sanitation, and hygiene practices.

### Caregiver level of education

Maternal education has been reported to be the best predictor of prompt malaria treatment in children under five in some countries in a multi-country analysis using national household survey data [14]. Three quarters of the respondents of this survey had not attended any formal school. This percentage is very similar to what was reported from a different region of Guinea [15]. This is much higher than what is reported from richer countries, such as South Africa, Cabo Verde and Equatorial Guinea [6, 16, 17]. There was no statistically significant association between level of education and malaria knowledge or bed net usage in this study. This could be because almost three-quarters of the survey respondents did not have any formal education.

### Disease burden

Almost half the children under the age of five were found to have had malaria and diarrhoea in their lifetimes as reported by their caretakers. In contrast, a desk review of ministry of health records in Conakry revealed malaria prevalence less than 10% in children under five between 2009 and 2012 [18]. The difference could be due to the fact that this survey was in a rural area which tends to have higher malaria prevalence compared to urban areas. Malaria prevalence in children under 5 has been reported to be between 20 and 40% in countries as varied as Ghana, Democratic Republic of Congo, Uganda, and Burkina Faso [19–21]. These data show the enormity of the malaria disease burden among children under 5 in sub-Saharan Africa.

Almost three quarters of the children had been taken to a health facility in the previous 6 months. More than half the child visits to a health facility were for preventive care such as routine checkups and vaccination. This is an encouraging finding that reveals the tremendous opportunity to provide routine preventive healthcare and health promotion counseling to young children and their caregivers by healthcare providers even in a rural setting.

### Caregiver gender

There was no significant difference in malaria knowledge between male and female caregivers, as well as different levels of education across all variables. Lack of significant difference in malaria knowledge between male and female caregivers could have been due to the low number of male caregivers in this survey. This contrasts with what has been reported from Bangladesh where level of education and gender were significant determinants of malaria knowledge and practice [5]. This could be due to differences in education levels between the two study populations. Also, since radio was reported as the most trusted source of -which makes it more likely that everyone in the community had equal access to this information regardless of gender or level of education-it is conceivable that malaria knowledge was equally distributed among the study population.

### Knowledge of malaria

Almost four-fifths of those surveyed reported mosquitoes as one cause of malaria, although this proportion halved when using the more stringent criterion of only mosquitoes being the cause of malaria. This finding is similar to what has been reported from other countries [7, 22]. However, this high percentage was in contrast to the 19% reported by a 2014 survey done in a different region of Guinea [15]. The high percentage of respondents who had knowledge of the cause of malaria is a heartening sign since lack of knowledge was previously found to be a major barrier to malaria prevention [23]. Fever was the most common symptom associated with malaria by study participants; this was similar to what has previously been reported [17].

### Bed net

A large percentage (86%) of the survey respondents had received a bed net during national campaigns. It is important to note thatthere was no statistically significant association between receiving information on malaria prevention and sleeping under a bed net. A previous analysis of post-bed net-campaign surveys from Nigeria found significant increase in bed net usage after behavioural change communication through mass media [24]. Secondary analysis of Nigerian Malaria indicator survey showed that topic-specific social and behavioural change communication improved the use of ITNs among children [25]. This information suggests that distributing long-lasting insecticide-treated mosquito nets through mass campaigns should be accompanied by an educational component. Receiving a free bed net was the strongest predictor of sleeping under one. Distribution of free bed nets has also been shown to increase its usage and decrease inequity in bed net ownership in Northern Nigeria [26]. A recent cross-sectional hospital-based survey in Guinea found significantly higher odds of malaria among pregnant women who had not regularly used ITNs [27]. Bed net ownership was 55% and their use was 79% in a survey from Forecariah district of Guinea [15]. The difference in ownership and use of bed nets from the two Guinea surveys could be attributed to the study year, geography, and culture. This survey was done 5 years after the survey in Forecariah [15]*,* and there were two LLIN mass campaigns in the years intervening the two surveys. Interestingly in South Africa, a country that was in the malaria elimination phase in 2016, 99% of respondents correctly identified the cause of malaria, but bed net use was reported as 2% during a survey [16]. Cabo Verde- another elimination country where 88% of survey respondents knew the cause of malaria and 97% had heard of mosquito nets-also showed a low 19% bed net use [6]. It is possible that as malaria prevalence decreases and countries move towards elimination, fewer people use bed nets as a method of malaria prevention. A study from Ghana, a richer country compared to Guinea, found no significant association between ITN use and malaria infection among children, except among children whose mothers had at least a secondary education [20]. Television was also found to be the best strategy to convey malaria education in Ghana [20]. These differences between countries show that the most effective malaria prevention and control approaches vary depending on local malaria prevalence [6]. Because it is often reported, that individuals access health information through radio and television, malaria education can be effectively delivered through these platforms [5, 6]. Threre was a statistically significant association between correctly identifying mosquitoes as the source of malaria and use of bed net the night before the survey. Since bed net usage is a major intervention to decrease malaria incidence, this underscores the importance of malaria knowledge in improving bed net usage and thus reducing its burden in Guinea.

### Water, sanitation and hygiene

A recent study concluded that water and sanitation conditions are associated with the risk of malaria among children under five years old, and the evidence this study supports these findings [9]. There was a statistically significant difference in caretaker hand washing frequency between children that ever had and had not had malaria. This relationship was also seen for reported diarrhoeal illness. It would be interesting to further investigate the relationship between drinking water source and malaria as well as diarrhoea risks because the majority of participants obtained their water from a borehole/hand pump. These are relatively clean sources of water that do not create stagnant water areas for mosquitos to thrive nearby.

### Healthcare access and practices

Approximately 40% of respondents sought care from government health posts while 40% sought care from both government health posts as well as traditional healers. In rural Burkina Faso, 72% of children with malaria received modern treatment while 18% received traditional treatment [19]. Local perception of disease was also found to affect treatment choice in Burkina Faso [11]. A similar number of respondents sought care from village doctors and drugstores in Bangladesh, which demonstrates the important roles played by traditional and informal healthcare providers in diverse developing countries [5].

More than half the survey respondents thought malaria treatment or consultations were not free at the level of the government health posts. This is a cause for concern as malaria treatment is meant to be free at government facilities due in part by support from international donors.

Approximately half of the participants indicated that it takes them over an hour to reach a health facility. Forty-six and 39% of the respondents waited one and two days, respectively before seeking medical care for illness. A similar delay to seek care was also reported from Bangladesh [5]. A previous KAP survey in a different area of Guinea found that seeking treatment in a formal health facility was dependent on socioeconomic status [15]. Initial treatment at home and waiting for more than 24 h was also seen in Equatorial Guinea [17]. Logistical obstacles and reliance on traditional remedies have been cited as major barriers to malaria treatment in a systematic review [23]. Almost all participantsindicated that malaria is a serious illness in adults and in children. This was similar to what was reported from Swaziland [7].

A large percentage of respondents preferred injectable malaria treatment as they considered it to be stronger and faster in action. This is another cause for concern since injections can cause complications such as infections and abscesses as well as necessitate proper disposal of sharps. This curious practice of injectable malaria treatment has previously been reported from Guinea by a much earlier study, showing the persistence of such treatment behaviour across more than two decades [28]. A 2016 cross-sectional survey in Guinea found that Artemisinin-based combination therapy (ACT) was prescribed for 84% of uncomplicated malaria cases even though almost a third of the malaria cases were diagnosed clinically without laboratory confirmation [29].

### Information source

Radio was the most cited and trusted source of information in this survey. Television was cited as the most common source of malaria information in the much richer Cabo Verde [6]. Media was also cited as the most common source of information on malaria behavioural change communication in Nigeria [24]. Our data as well as results from these countries show that the media plays an important role in disseminating health information. Efforts to inform and educate Guineans on malaria prevention must continue using radio as a channel for communication.

### Study limitations

This study results must be interpreted in light of its limitations. The survey was conducted in 2016 and some of the findings may no longer be relevant or accurate. For example, some of the perceptions about malaria and diarrhoea may have changed due to media and health campaigns. Generalizing the survey findings to the rest of Guinea must also be done with caution as Guinea is a geographically and ethnically diverse country. Some readers may note that the authors have reported an abundance—some might even say an overabundance—of observations from the survey without a narrow focus or depth. There was now deep analysis by looking for complex associations and adjusting for confounders. This was an old-fashioned study of the kind that was not planned to test specific hypotheses, but rather to gather basic data and generate some leads for further research. The authors were primarily interested in painting the widest possible picture of knowledge, attitudes and practices around malaria and diarrhoeal illness in the study area rather than diving deep into contributing factors and associations. The raw data and survey questions have been made available to the scientific community to conduct further analyses of their own or design future studies.

## Conclusion

Guinea was struggling with reducing malaria mortality even before the arrival of COVID-19. The current target of achieving pre-elimination by 2022 will become even more challenging in this time when national malaria control programmes worldwide are predicting serious setbacks from interruption of efforts. This study documents a detailed collage of knowledge, attitudes and practices among members as they relate to malaria in a rural Guinean community with a high malaria burden. This was a community where most caregivers of children under five were women with very little formal education. Despite this lack of education, they knew the causes of malaria and diarrhoea, and they were taking their children for preventive care to local health posts. Malaria and diarrhoea were considered serious illnesses and were treated at both formal, informal, and traditional healthcare providers. There was high usage of bed nets and an associated contribution to reducing malaria. We have identified gaps in current knowledge as well as suggested how to target those gaps. Comparing thesefindings with those of others from Guinea as well as from other countries makes it clear that effective interventions to control and prevent malaria will need a detailed understanding of local facts. This study adds valuable information for policy makers and practitioners who design and implement malaria control and prevention measures in Guinea.

## Data Availability

All data generated or analysed during this study are included in this published article [and its supplementary information files].
